# Editorial: Recent advancements on the development and ripening of Mediterranean fruits and tree crops

**DOI:** 10.3389/fpls.2023.1256315

**Published:** 2023-07-26

**Authors:** Ashraf El-kereamy, Marco Caruso, Carolina A. Torres, Ana M. Cavaco

**Affiliations:** ^1^Department of Botany and Plant Sciences, University of California, Riverside, Riverside, CA, United States; ^2^Council for Agricultural Research and Economics (CREA), Research Centre for Olive, Fruit and Citrus Crops, Acireale, Italy; ^3^Department of Horticulture, Tree Fruit Research and Extension Center, Wenatchee, Washington State University, Wenatchee, WA, United States; ^4^Center of Electronics, Optoelectronics and Telecommunications (CEOT), University of Algarve, Faro, Portugal

**Keywords:** fruit tree, olive, grapes, citrus, avocado, transcriptome, light manipulation, fruit quality

The Mediterranean basin and other Mediterranean-type ecosystems (MTE) are home to many tree crops domesticated and adapted well to their environment. Several of them present specific development and ripening traits that challenge established models. Climate changes that are occurring in the Mediterranean area and in other MTE tends to aggravate the already irregular rainfall and temperature patterns, posing detrimental outcomes on crop performance, productivity, and changes in fruit ripening. With these climate changes, one would expect changes in the fruits and tree crops components growing in these ecosystems. Currently, we are experiencing a tremendous advance in the technology that allows researchers to study in-depth the basic phenomenon and find significant novel data to establish guidelines for new cultural practices, breeding programs, and variety selection that can better adapt to the changing conditions. The goal of this Research Topic was to highlight recent studies on the anatomical, physiological, metabolomic, and genomic processes occurring throughout the development and ripening of fruits and tree crops grown in the Mediterranean Basin and MTE, from field until postharvest. Since many of them are perennial species, they are subjected to adverse environmental conditions throughout their entire life cycle. Thus, the effect of cultural practices, varying environmental factors, as well as the impact of the various stresses on the performance of these tree crops were also acknowledged.

Rising temperature and unpredictable extreme weather events are some of the characteristics of the new climate change scenario that challenges production (and quality) of some of the Mediterranean crops growing worldwide. For instance, the production of premium wine and table red grapes (*Vitis vinifera* L.) is being affected by global warming. Anthocyanin is the pigment responsible for the red color in grapes, and its biosynthesis is regulated by various factors such as cultural practices, environmental conditions, and plant hormones. Afifi et al. concluded that manipulating the light spectrum and applying silicon in combination with the ethephon treatment could be used in table grape to improve ethylene-induced anthocyanin accumulation and coloration. These treatments were able to increase the expression of *flavonoid-3-O-glucosyltransferase* (UFGT) and Peroxidase dismutase (POD), critical in anthocyanin biosynthesis The study provided evidence of the need for multi-approaches and the importance of the antioxidant system to cope with the impact of climate change on red grapes.

Lacking water in the soil due to drought and warmer conditions, as well as the use of low-quality water cause the accumulation of salts and negatively impact tree growth and productivity, posing a significant challenge to tree crops such as olive (*Olea europea* L.) in the Mediterranean basin. Tadic et al. showed the variation in the response of two well-known olive cultivars compared to a wild olive genotype (WOG) known as “Perišićeva mastrinka” under salt-stress conditions. Both the osmotic (300 mM mannitol) and the ionic (150 mM NaCl) components of salinity were accounted for. It appears that both stressors affected the growth of cv. Leccino (salt-resistant), which accumulated less potassium compared to cv. Koroneiki (salt-sensitive) and WOG. Otherwise, the growth parameters of the cv. Koroneiki and WOG of different ages decreased only in response to the osmotic stressor, which also had severe physiological and biochemical effects on WOG. These data suggest that WOG resilience to salinity is associated with its large leaf capacity for Na+ and Cl− accumulation, K+ retention, and its adaptable antioxidative mechanisms. Regarding the latter, the absence of lipid peroxidation along with suppressed GPOX activity suggests that some other enzymes, such as catalase and ascorbate peroxidase, could be involved in the detoxification of H_2_O_2_. The results are promising and could be used to obtain new olive cultivars with better resilience to soil salinity.

In another study, Martins et al. investigated the drought tolerance mechanisms of four different genotypes of the strawberry tree (*Arbutus unedo* L.) from various geographic origins, a plant species with high ecological relevance in southern European forests, and several economical applications. This comprehensive study assessed several eco-physiological and biochemical parameters, and the metabolomic profiles on plants from those genotypes, under different water regimes (plants watered to 70% and 18% field capacity) and a recovery assay. Overall, the study highlights the importance of the genotype as a major selection criterion for resistant plants to drought and provides empirical knowledge of the metabolic response involved. Indeed, it was found that the different genotypes showed contrasting drought tolerance and recovery ability. This was associated with different levels of physiological control, with a trade-off between transpiration water loss and CO_2_ assimilation, and the differential accumulation of more than 500 metabolic features, including abscisic and salicylic acids, for the genotype with the best performance under drought, and the fastest recovery after the stress interruption. Additionally, it was found that the genotype with the most efficient strategy under drought conditions also presented several metabolites in higher baseline concentrations (control groups). The involvement of phenolics in plant response mechanisms under drought was also hypothesized, which could shed light on the metabolic pathways involved in plant response to water stress. Since most strawberry tree orchards are installed on marginal lands where plants usually face severe drought, selecting plants that can better cope with water restriction is critical, and a better understanding of the tolerance mechanisms is required. This study contributed significantly not only for that, but also provided a basis for marker assisted breeding towards drought resistance.

The study of varietal diversity of tree crops significantly contribute to determining fruit quality and nutritional value, and breeding value, to improve consumer acceptance and market share. For instance, Zacarías-García et al. characterized carotenoid metabolism of two lycopene-pigmented sweet orange (*Citrus sinensis* (L.) Osbeck) varieties, Kirkwood Navel and Ruby Valencia, which are two spontaneous bud mutations of Palmer Navel and Olinda Valencia, respectively. Interestingly, the altered carotenoid profiles and accumulation in Kirkwood and Ruby fruits are not explained by differences in the transcriptional profile of 26 genes related to carotenoid metabolism, including those involved in biosynthesis, catabolism, and other processes related to carotenoid accumulation. It is worth noting that the mutation is not only manifested in the fruit, but also other carotenogenic tissues of the mutant plants, such as the bark tissue, with different consequences in the carotenoid profile. Overall, the carotenoid composition in the red-fleshed mutants suggests a partial blockage of the lycopene β-cyclization in the carotenoid pathway, resulting in a high accumulation of carotenes upstream of lycopene (phytoene, phytofluene), δ-carotene, and a reduced flow to downstream xanthophylls and ABA. However, the possible mechanisms regulating lycopene accumulation in the pulp of the two citrus mutants remains unknown and need further investigation.

Muscadine grape (*Muscadine rotundifolia* Michx.) is receiving greater attention due to their tolerance to several diseases that cause extensive economic losses in bunch grapes and their enhanced nutraceutical value as a result of the higher accumulation of bioactive phenolics compared with other grape species. Aiming to understand the crucial, but still unknown gene network that can cause an intervarietal difference in phenolic (TPC)/flavonoid (TFC) accumulation in muscadine grape Ismail et al. investigated the molecular events associated with polyphenolic accumulation and antioxidant activity in two genotypes (C5 and C6) during berry development. Results indicated that transcriptional modulation of a large number of genes occurs during berry development, the transcriptomic profile changes being more pronounced at the véraison (V) stage. Despite the downregulation pattern of gene expression dominating the upregulation, the C5 genotype maintained higher expression levels, suggesting a potential association with the accumulation of bioactive phenolics. Comparative transcript profiling identified 94 differentially expressed genes with potential relevance in regulating fruit secondary metabolism, including 18 transcription factors and 76 structural genes. The genes encoded key enzymes involved in the modification reactions of polyphenolics biosynthetic pathway, including hydroxylation, methylation, and glycosylation, and were more abundant during the V stage of C5 genotype than C6, defining the high accumulation of bioactive phenolic/flavonoid derivatives in C5. The current investigation provides the first extensive record for mRNA expression profiling in parallel with the changes in TPC/TFC accumulation and antioxidant activity throughout muscadine berry development, which have important implications for the breeding of muscadine grape varieties with improved phenolic content and antioxidant activity, providing potential health benefits for consumers.

Another study included in the Research Topic emphasized the role of genotype × environment interaction and the need to investigate cultivar adaptation to different geographical areas to define the best ideotypes for cultivation. Salameh et al. evaluated the postharvest maturity indices of commercial avocado (*Persea americana* Mill.) varieties grown in different regions in Lebanon. The researchers examined dry matter (DM), oil content (OC), fruit firmness, titratable acidity (TA), total soluble solids (TSS/Brix), and fruit weight over three harvest stages for seven different avocado varieties: Hass, Lambhass, Ettinger, Fuerte, Pinkerton, Reed, and Horshim. The varieties were grown at different altitudes ranging from 50 to 400 m in seven regions in Lebanon. The study found that DM and OC had a high linear correlation over the different harvest stages. During the late harvest stage, fruit firmness showed a negative correlation with TSS. The Reed variety had the lowest oil content % and dry matter %, while the Fuerte variety had the highest oil content % and dry matter %. Overall, the study showed no correlation between fruit weight and location’s altitude, although there were differences between cultivars. The study’s findings provide important information for determining the best timing to harvest avocados to achieve the best edible characteristics and meet export standards.

In conclusion, the articles published in this Research Topic provide valuable up-to-date information about novel technologies and advancements in Mediterranean fruits and tree crops research.

## Author contributions

All authors listed have made a substantial, direct, and intellectual contribution to the work and approved it for publication.

